# Atypical Trait Inferences From Facial Cues in Alexithymia

**DOI:** 10.1037/emo0000066

**Published:** 2015-04-13

**Authors:** Rebecca Brewer, Fredrika Collins, Richard Cook, Geoffrey Bird

**Affiliations:** 1 MRC Social, Genetic, & Developmental Psychiatry Centre, Institute of Psychiatry, King’s College London; 2 School of Medical Education, King’s College London; 3 Department of Psychology, City University London; 4 MRC Social, Genetic, & Developmental Psychiatry Centre, King’s College London, and Institute of Cognitive Neuroscience, University College London

**Keywords:** alexithymia, facial emotion, character traits, trustworthiness, attractiveness

## Abstract

It is often difficult to distinguish strangers’ permanent facial shapes from their transient facial expressions, for example, whether they are scowling or have narrow-set eyes. Overinterpretation of ambiguous cues may contribute to the rapid character judgments we make about others. Someone with narrow eyes might be judged untrustworthy, because of strong associations between facial anger and threat. To test this hypothesis, we investigated the trait judgments made by individuals with severe alexithymia, associated with impaired recognition of facial emotion. Consistent with the hypothesis, alexithymic participants demonstrated reduced interrater consistency when judging the character traits of unfamiliar faces, and the presence of subtle emotions. Nevertheless, where alexithymics perceived, or misperceived, emotion cues, the character traits inferred thereafter were broadly typical. The finding that individuals with developmental deficits of emotion recognition exhibit atypical attribution of character traits, confirms the hypothesis that emotion-recognition mechanisms play a causal role in character judgments.

Humans frequently judge the character of others based solely on their facial shape. These trait inferences are far from trivial: The initial impressions we form about someone’s character may determine if, and how, we interact with that person. Although our first impressions are not always accurate (Olivola & Todorov, 2010), different raters draw highly consistent inferences, even when judging static images of actors exhibiting neutral facial expressions (Said, Haxby, & Todorov, 2011; Todorov, Pakrashi, & Oosterhof, 2009; Willis & Todorov, 2006). Moreover, trait inferences are available extremely quickly. Stable judgments are made within 100 ms of the appearance of a novel face (Willis & Todorov, 2006), and interrater agreement is above chance after only 33 ms (Todorov et al., 2009).

Trait judgments made about emotionally neutral models may be a product of neurocognitive mechanisms adapted for emotion recognition (Said et al., 2011; Zebrowitz & Montepare, 2008). It is not always easy to distinguish a stranger’s permanent facial shape from their transient facial expressions. According to the emotion-overgeneralization hypothesis, permanent facial features that resemble subtle facial emotions may provoke inferences in line with those provoked by the corresponding emotional expression (Montepare & Dobish, 2003; Zebrowitz & Montepare, 2008). For example, lower eyebrows—a common feature of facial displays of anger—may cause an individual to be perceived as dominant.

Consistent with the emotion-overgeneralization hypothesis, the trait inferences drawn by observers correspond closely with their reading of the model’s facial emotion (Said et al., 2011). For example, emotionally neutral models perceived to be happy or angry are also likely to be judged as trustworthy and aggressive, respectively (Oosterhof & Todorov, 2008; Said, Sebe, & Todorov, 2009). Recognition of facial emotion (e.g., Adolphs, 2002; Hariri, Tessitore, Mattay, Fera, & Weinberger, 2002) and inference of character traits (Engell, Haxby, & Todorov, 2007; Winston, Strange, O’Doherty, & Dolan, 2002) are thought to recruit similar neural substrates, notably the amygdala. Common mechanisms are also suggested by the occurrence of cross-adaptation between traits and emotions. Periods of psychophysical adaptation to angry or happy faces make subsequently viewed faces appear more and less trustworthy (Engell, Todorov, & Haxby, 2010). The ability to recognize facial emotion (Duchaine, Parker, & Nakayama, 2003) and infer character traits typically (Rezlescu, Susilo, Barton, & Duchaine, 2014; Todorov & Duchaine, 2008) is often preserved in cases of prosopagnosia, despite impaired recognition of facial identity.

If trait inferences depend on neurocognitive mechanisms adapted for the recognition of facial emotion, individuals who exhibit deficits of emotion recognition should also draw atypical trait inferences (Adolphs, Tranel, & Damasio, 1998). We sought to test this hypothesis in the present study by comparing the trait inferences made by individuals with alexithymia with those made by typical control individuals. Alexithymia is a nonclinical condition characterized by a reduced ability to identify and describe one’s own emotion; for example, individuals with alexithymia might know that they are experiencing an emotion, but be unaware whether that emotion is sadness, anger or fear (Nemiah, Freyberger, & Sifneos, 1976). Crucially however, the condition results in impaired recognition of others’ emotions (Cook, Brewer, Shah, & Bird, 2013; Parker, Prkachin, & Prkachin, 2005; Prkachin, Casey, & Prkachin, 2009). Alexithymia is thought to be the product of developmental dysfunction of, or reduced connectivity between, limbic structures implicated in the subjective experience of emotion and affect recognition (e.g., Bird et al., 2010; FeldmanHall, Dalgleish, & Mobbs, 2013; Ihme et al., 2013; Moriguchi et al., 2007).

## Experiment 1

In our first experiment, we sought to verify that our sample of alexithymic individuals exhibited impaired recognition of facial emotion. Fifteen individuals with alexithymia (*M*_age_ = 28.67, *SD*_age_ = 14.91, 10 women) and 19 typical control individuals (without alexithymia; *M*_age_ = 22.68, *SD*_age_ = 3.13, 11 women) participated in the study. No participant had a previous or current diagnosis of any developmental or psychological disorder. Sample size was determined by the availability of alexithymic participants; 650 individuals were screened for alexithymia using the Toronto Alexithymia Scale (Parker, Taylor, & Bagby, 2003). Of these individuals, 15 with alexithymia were identified, and agreed to participate in the study. All members of the alexithymia group met the standard criterion for the presence of alexithymia (score > 60). A control group was identified through selection of individuals so that groups would be matched according to demographic variables. The alexithymia and control groups were matched according to IQ, *t*(32) = 1.63, *p* = .112, assessed by the Wechsler Abbreviated Scale of Intelligence (Wechsler, 1999), age, *t*(32) = 1.60, *p* = .131, and gender, χ^2^(1) = .64, *p* > .250. All participants took part in all three experiments. Experiment 3 followed Experiment 2 for every participant, but the order of this pair of experiments and Experiment 1 was counterbalanced.

**Figure 1 fig1:**
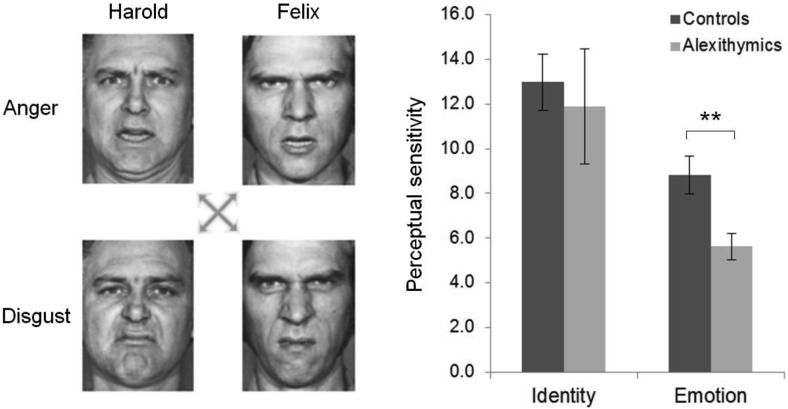
The stimuli in Experiment 1 were drawn from two complementary morph continua blending Harold displaying anger with Felix displaying disgust, and Harold displaying disgust with Felix displaying anger (left). When asked to discriminate the identity and emotion of the stimulus images, the alexithymic participants showed reduced sensitivity to changes in facial emotion, but broadly typical sensitivity to changes in identity (right). Face stimuli taken from Karolinska Database. Error bars indicate ±1 *SEM.* * *p* < .05; ** *p* < .01; *** *p* < .001.

The stimuli used in the first experiment were 14 grey-scale images comprising two complementary cross-morph continua, blending (a) “Harold” displaying anger with “Felix” displaying disgust, and (b) “Harold” displaying disgust with “Felix” displaying anger. The endpoints of the continua were images taken from Ekman and Friesen’s (1976) Pictures of Facial Affect, Identities M4 and M6 (Figure 1). The images were morphed using Morpheus Photo Morpher Version 3.11 (Morpheus Software, Indianapolis, IN). The 14 images represented points along the morph continua corresponding to intensities between 20% and 80% of each attribute, varying in 10% intervals. Facial images were cropped to remove external features, and subtended 6° × 8° when viewed at a distance of 60 cm.[Fig-anchor fig1]

Participants completed 10 blocks of 28 experimental trials, preceded by eight practice trials. Each trial began with a fixation point (1500 ms), followed first by the presentation of a morph stimulus (800 ms), and then by a prompt to attribute either identity or emotion (i.e., “disgust or anger?” or “Harold or Felix?”). Each cross-morph stimulus appeared twice per block, followed once by an identity prompt, and once by an emotion prompt. Attribution type was interleaved within blocks, thereby forcing participants to attend to both identity and expression on any given trial. The identity and emotion labels were presented at the start of the practice and experimental tasks and between blocks.

For the emotion and the identity tasks, the percentage of one attribute (e.g., Harold) in each stimulus was plotted against the probability of making that attribution (i.e., responding “Harold”) to that stimulus, for each participant. Psychometric functions were estimated in Matlab (MathWorks, Natick, MA) using the Palamedes toolbox (Prins & Kingdom, 2009) by fitting cumulative Gaussian (S-shaped) functions to this data for each individual. Perceptual sensitivity was inferred from the slope of the psychometric function, defined as the standard deviation of the underlying (symmetric) Gaussian distribution, subject to an inverse transform to normalize parameter estimates. The experimental programs used in all experiments were written and presented in Matlab with the Psychophysics Toolbox (Brainard, 1997; Pelli, 1997).

Experiment 1 assessed the recognition of disgust and anger, as they are the emotions that correlate most strongly with the judgments of trustworthiness, aggressiveness, attractiveness, and intelligence used in Experiment 2. It should be noted, however, that this disgust–anger recognition task and an identical task assessing the recognition of surprise and fear have previously been employed to demonstrate significant correlations between alexithymia and emotion recognition (Cook et al., 2013), supporting previous findings of recognition impairment across a range of emotions in alexithymic individuals (Parker et al., 2005; Prkachin et al., 2009).

Consistent with previous reports of impaired emotion recognition in this population, the alexithymia group exhibited lower sensitivity (*M* = 5.64, *SD* = 2.28) than the control group (*M* = 8.83, *SD* = 3.68) to changes in facial emotion, *t*(32) = 2.937, *p* = .003, CI [1.29, 6.32] (Figure 1), and perceptual sensitivity to emotion correlated significantly with individual differences in alexithymia severity, *r* = −.371, *p* = .015. Conversely, the alexithymic (*M* = 11.89, *SD* = 9.96) and control (*M* = 12.97, *SD* = 5.48) individuals did not differ in their sensitivity to changes in facial identity, *t*(32) = −.195, *p* = .846, and identity sensitivity did not correlate with alexithymia severity, *r* = −.073, *p* = .681.

## Experiment 2

In our second experiment, we sought to examine how alexithymia affects the consistency of trait and attractiveness judgments made about emotionally neutral models. The stimuli used in Experiment 2 were 40 grey-scale images depicting emotionally neutral models taken from the Karolinska Directed Emotional Faces Database (Lundqvist, Flykt, & Ohman, 1998). The external contour of each face was visible, but hair and any external features were removed. Stimuli were selected based on previous ratings (Lundqvist et al., 1998) to sample a representative range of values for each trait being investigated. Images subtended 9° × 11° when viewed at a distance of 60 cm.

Four judgments were made about each image (trustworthiness, aggressiveness, intelligence, and attractiveness). Ratings were made on a scale ranging from 1 (*Not at all*) to 9 (*Extremely*). Each trial presented a face stimulus together with a prompt to rate the image on a given dimension (e.g., “How trustworthy is this person?”). Instructions emphasized that participants should base their decisions on their first impressions, but no time limit was imposed. Stimuli remained present until a response was recorded. Each image was rated twice on each dimension, yielding a total of 320 trials. The two judgments given to each stimulus were averaged and analyses conducted on the resulting distributions. Participants completed four blocks of 80 trials.

**Figure 2 fig2:**
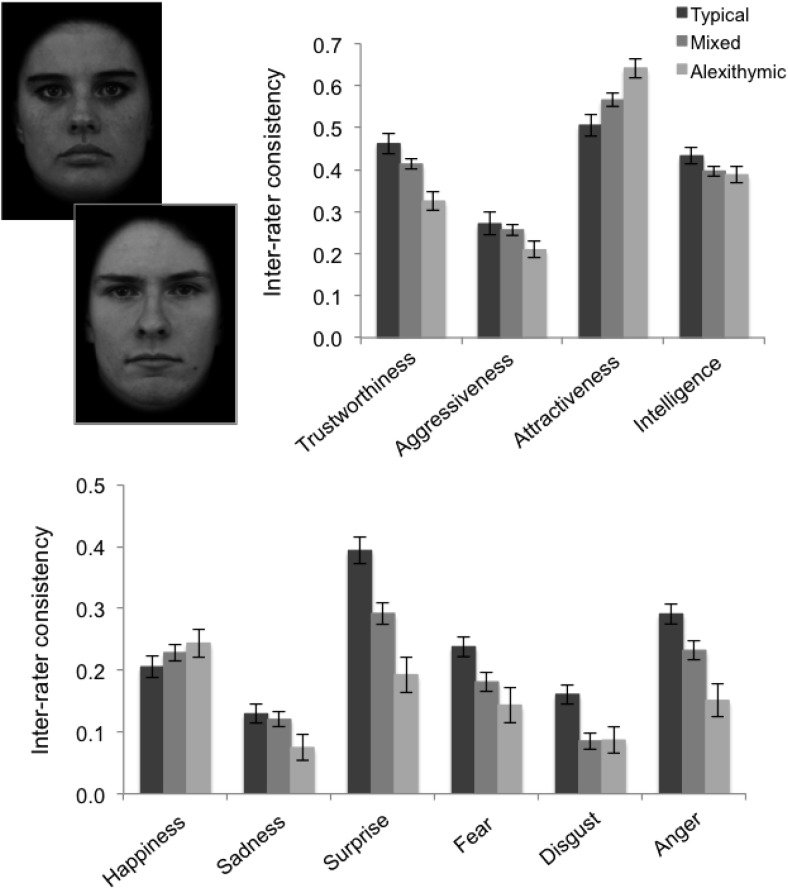
Participants made character and social judgments (Experiment 2), and emotion judgments (Experiment 3), about the same emotionally neutral models (top left). In Experiment 2, the alexithymic individuals exhibited lower interrater consistency than typical individuals when judging trustworthiness, but greater consistency when judging facial attractiveness (top right). In Experiment 3, the alexithymic participants also exhibited poorer interrater consistency when detecting subtle facial emotions, notably surprise and anger (bottom). Face stimuli taken from Karolinska Database. Error bars indicate ±1 *SEM*.

For each judgment, an index of interrater consistency was derived by calculating the correlations between ratings for every possible pair of participants (Todorov & Duchaine, 2008; Todorov et al., 2009). Having subjected the raw pairwise correlations to Fisher *z* transformations, the consistency of each attribution was assessed by comparing the resulting distributions of the control–control pair, control–alexithymic (mixed) pair, and alexithymic–alexithymic pair correlations. Figure 2 demonstrates that interrater consistency was highest for the control pairs, reduced for mixed pairs, and lowest of all for the alexithymic pairs.[Fig-anchor fig2]

While participant groups were statistically matched in terms of gender, the proportion of females in the alexithymia group was higher (67%) than that in the control group (58%) and several studies have demonstrated an effect of gender on judgments relating to facial emotion, including trait inferences (McClure, 2000; Mehu, Little, & Dunbar, 2008). Gender was therefore included as a covariate in an ANCOVA with judgment (aggressiveness, trustworthiness, attractiveness, intelligence) as a within-subjects factor and pair type (control, mixed, alexithymic) as a between-subjects factor. The main effect of gender was, indeed, significant in this analysis, *F*(1, 557) = 4.00, *p* = .046, η^2^ = .007, and gender interacted significantly with the effect of trait, *F*(3, 1671) = 2.81, *p* = .038, η^2^ = .005. The ANCOVA revealed a significant main effect of judgment, *F*(3, 1671) = 71.96, *p* < .001, η^2^ = .114. Interrater consistency was significantly higher for attractiveness than for all other judgments (all *p*s < .001), and significantly lower for aggressiveness than for all other judgments (all *p*s < .001), regardless of pair type. Crucially, the analysis also revealed a significant Pair Type × Judgment interaction, *F*(3, 861) = 18.49, *p* < .001, η^2^ = .037. The interrater consistency of trustworthiness provided by the alexithymic pairs (*M* = .33, *SD* = .24, CI [.28, .37]) was significantly lower than that of the mixed pairs, *M* = .41, *SD* = .25, CI [.39, .44], *t*(338) = 3.25, *p* = .001, which was in turn significantly lower than that of the control pairs, *M* = .46, *SD* = .25, CI [.43, .50], *t*(454) = 2.04, *p* = .040. Interrater consistency of aggressiveness ratings was significantly lower in alexithymic pairs, (*M* = .21, *SD* = .27, CI [.16, .26]) than in control pairs, *M* = .27, *SD* = .26, CI [.23, .31], *t*(274) = 2.21, *p* = .028. For intelligence ratings, there was a trend for control pairs (*M* = .43, *SD* = .21, CI [.40, .47]) to demonstrate higher interrater consistency than alexithymic pairs, *M* = .39, *SD* = .22, CI [.35, .43], *t*(274) = 1.81, *p* = .069. Strikingly, however, the alexithymic pairs (*M* = .64, *SD* = .29, CI [.59, .70]) exhibited greater interrater consistency than mixed pairs, *M* = .57, *SD* = .29, CI [.53, .60], *t*(388) = 2.30, *p* = .021, who demonstrated greater interrater consistency than control pairs, *M* = .51, *SD* = .29, CI [.46, .55], *t*(454) = 2.14, *p* = .030, in their ratings of facial attractiveness.

## Experiment 3

Our third experiment investigated how alexithymia affects the consistency of emotion judgments made about emotionally neutral models. The 40 stimuli employed in Experiment 3 were identical to those used in Experiment 2. Participants rated each stimulus according to the extent to which it depicted six subtle emotions (happiness, sadness, disgust, anger, surprise, and fear) on a scale from 1 (*Not at all*) to 9 (*Moderately*). Stimuli again remained present until response. Each image was rated once for each emotion, yielding a total of 240 trials. Correlations were calculated for each emotion, for every possible pair of participants within and between groups, and subjected to Fisher *z* transformations. Thereafter, the consistency of each attribution was assessed by comparing the resulting distributions of control, mixed, and alexithymic pairs. Interrater consistency was again highest for the control pairs, reduced for mixed pairs, and lowest of all for the alexithymic pairs (see Figure 2).

An ANCOVA including gender was initially performed as above, but in this analysis, gender was not a significant predictor of judgments. ANOVA was therefore employed, with emotion (happiness, sadness, disgust, anger, surprise, fear) as a within-subjects factor and pair type (control, mixed, alexithymic) as a between-subjects factor, and revealed a significant main effect of emotion, *F*(5, 2785) = 48.98, *p* < .001, η^2^ = .081. Interrater consistency was significantly higher for surprise than for all other emotions (all *p*s < .002), whereas the consistency of sadness and disgust ratings was significantly lower than that of the other four emotions (all *p*s < .001). Importantly, a significant main effect of pair type, *F*(2, 557) = 16.05, *p* < .001, η^2^ = .054, indicated that the emotion ratings of the alexithymic pairs (*M* = .15, *SD* = .11, CI [.12, .17]) were less consistent than those of the mixed pairs, *M* = .19, *SD* = .12, CI [.18, .21], *t*(287) = 3.18, *p* = .005, which, in turn, were less consistent than the control pairs, *M* = .24, *SD* = .12, CI [.22, .26], *t*(287) = 3.18, *p* < .001.

Pair type also interacted significantly with emotion, *F*(10, 2790) = 4.83, *p* < .001, η^2^ = .017. Alexithymic pairs were less consistent than mixed and control pairs for sadness judgments, mixed: *t*(388) = 1.96, *p* = .047; control: *t*(274) = 2.20, *p* = .027; alexithymic pairs were less consistent than mixed pairs, who were less consistent than control pairs, for surprise and anger judgments, surprise: alexithymic vs. mixed, *t*(388) = 3.00, *p* = .003; mixed vs. control, *t*(454) = 5.58, *p* < .001; anger: alexithymic versus mixed, *t*(388) = 2.89, *p* = .004; mixed versus control, *t*(454) = 2.46, *p* = .014, while alexithymic and mixed pairs were less consistent than control pairs for fear and disgust judgments, fear: alexithymic vs. control, *t*(274) = 3.03, *p* = .003; mixed vs. control, *t*(454) = 2.37, *p* = .021; disgust: alexithymic versus control, *t*(274) = 2.70, *p* = .008; mixed versus control, *t*(454) = 3.52, *p* = .001.

**Figure 3 fig3:**
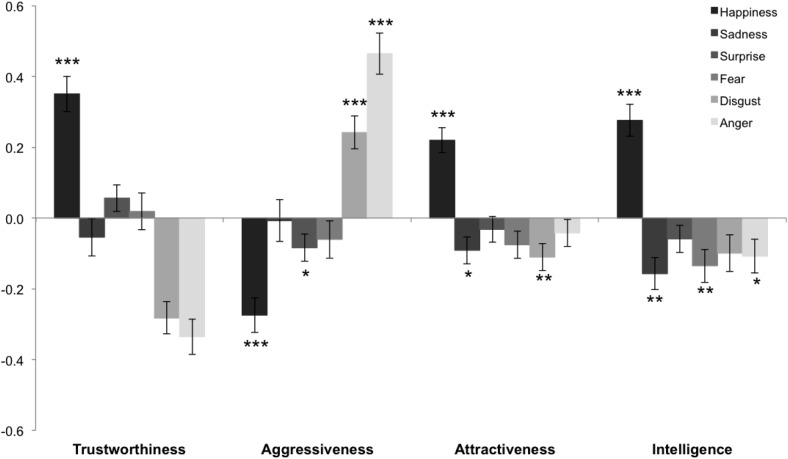
The propensity to read certain emotions into the emotionally neutral faces (Experiment 3) correlated with judgments made about those faces (Experiment 2). Typical and alexithymic participants demonstrated similar levels of association between emotion and trait inferences. Whether alexithymic individuals perceive, or misperceive, emotional cues, the character traits inferred thereafter appear broadly typical. Raw correlations were subject to Fisher’s *z* transformations and the resulting distributions were evaluated using one-sample *t* tests. Error bars indicate ±1 *SEM*. * *p* < .05; ** *p* < .01; *** *p* < .001.

To determine whether participants were basing their trait judgments on emotion cues, we calculated for each participant the simple correlations between the trait and emotion ratings given to each model. These correlations were subjected to a Fisher’s *z* transformation and the resulting distributions analyzed using ANOVA with emotion (happiness, sadness, disgust, anger, surprise, fear) and judgment (aggressiveness, trustworthiness, attractiveness, intelligence) as within-subjects factors and group (control, alexithymic) as a between-subjects factor. The analysis revealed a significant Trait × Emotion interaction, *F*(15, 480) = 24.41, *p* < .001, η^2^ = .433, confirming that different traits were inferred from the presence of different emotions. Faces judged low on happiness, and high on disgust and anger, were deemed to be aggressive and untrustworthy, whereas models perceived as happy were rated as attractive and intelligent (see Figure 3). The relationship between perceived emotion and trait judgments did not vary as a function of group, *F*(1, 32) < .01, *p* = .956, η^2^ < .001, suggesting that individuals with alexithymia draw inferences from emotion cues in the same way as control participants, despite exhibiting inconsistent attribution of facial emotion.[Fig-anchor fig3]

## Discussion

According to the emotion-overgeneralization hypothesis, trait judgments made about emotionally neutral models may be a product of neurocognitive mechanisms adapted for emotion recognition (Said et al., 2011; Zebrowitz & Montepare, 2008). Permanent facial features resembling subtle facial emotions such as low eyebrows or narrow-set eyes, may provoke inferences in line with those provoked by the corresponding emotional expression. The present study sought to test this hypothesis by determining whether individuals with alexithymia, a condition associated with impaired emotion recognition, exhibit atypical trait judgments. In our first experiment, alexithymic individuals demonstrated reduced sensitivity to subtle changes in facial emotion, confirming previous reports. In our second and third experiment, alexithymia was associated with reduced interrater consistency when participants judged the character traits (Experiment 2) and emotions (Experiment 3) of emotionally neutral models.

That individuals with developmental deficits of emotion recognition tend to draw atypical inferences (differing from control individuals) about the character of others suggests that mechanisms of emotion recognition play a causal role in trait judgments. These results therefore accord well with previous reports that trait inferences correlate with propensity to read certain emotions in faces (Oosterhof & Todorov, 2008; Said et al., 2011, 2009) and reports of atypical trait inferences in patients with acquired deficits of emotion recognition (Adolphs et al., 1998). The observation of atypical trait inferences in individuals with impaired emotion processing, but broadly intact face perception, confirms that emotion cues, based on underlying facial structure, contribute to trait inferences. Individuals with and without alexithymia demonstrated similar levels of association between emotion and trait inferences. One possibility is that, causally, emotion detection takes place before the inference of character traits, and individuals with alexithymia are selectively impaired at emotion detection. Hence, whether alexithymic individuals perceive or misperceive emotional cues, the character traits inferred thereafter are broadly typical.

It is interesting to consider whether the pattern demonstrated by alexithymic individuals of atypical emotion and trait inference, in the presence of typical associations between emotion and trait judgments, elucidates the reasons that particular emotion attributions prompt particular trait attributions. As some theorists have centered on semantic links between certain emotions and traits (e.g., anger and aggressiveness, see Said et al., 2009), other theorists have adopted an ecological approach, suggesting that cues to emotion provide valid cues to dominance and affiliation and therefore encourage approach or avoidance behaviors (e.g., Montepare & Dobish, 2003). Whereas the ecological approach explains some relationships between emotions and traits well, others are less well explained under this framework, such as the link between happiness and intelligence. Rather than happiness being a valid cue to intelligence, previous research has demonstrated a negative association between happiness and intelligence (Veenhoven & Choi, 2012). Future work investigating dominance and affiliation attributions in response to perceived emotion in individuals with alexithymia, as well as predictors of trait attributions in this population, may serve to shed light on the relative contributions of semantic associations and dominance and affiliation cues in determining emotion–trait correlations in this group.

Alexithymic individuals exhibited poor interrater consistency when judging trustworthiness, aggressiveness, and intelligence, while their ratings of attractiveness were more consistent than those of control individuals. The relationship between facial emotion and perceived attractiveness may differ from that hypothesized for character traits. Although perceived facial attractiveness may be based on structural attributes including symmetry, sexual dimorphism, and averageness (Rhodes, 2006; Thornhill & Gangestad, 1999), judgments may also be affected by emotion cues. For example, previous results suggest that smiling faces are perceived as more attractive (Mehu et al., 2008). Similarly, we observed that faces perceived as happy tended to be judged as more attractive. The absence of reliable cues to emotion may encourage individuals with alexithymia to base judgments of facial attractiveness on structural features such as symmetry alone. As individuals without alexithymia base their judgments on both structural and emotional information, the control population may therefore exhibit wider individual differences and reduced interrater consistency than the population of individuals with alexithymia.

The present results not only inform the study of normative trait inferences, but also have important implications for the study of social cognition in clinical disorders. It is well-established that alexithymia co-occurs with several clinical conditions, including autism spectrum disorder (ASD), schizophrenia, mood disorders, and eating disorders, and may be responsible for inconsistent reports of impaired emotion recognition in these populations (Bird & Cook, 2013; Cook et al., 2013). The present findings suggest that psychiatric patients with co-occurring alexithymia may not only exhibit impaired recognition of facial emotion, but may also draw unusual inferences about the character of others. This may contribute to the social difficulties faced by individuals with alexithymia: Although it is unlikely that judgments about others based on facial characteristics provide genuine information about that person, it is possible that making similar judgments to others is an important factor for one’s integration into social groups. If a particular individual is regarded by a social group as untrustworthy, for example, group acceptance may rely on a group member also concluding that this individual is untrustworthy (regardless of his or her objective trustworthiness). As many clinical disorders with co-occurring alexithymia, such as ASD, schizophrenia, and eating disorders, are associated with impaired social functioning (American Psychiatric Association, 2013; Caglar-Nazali et al., 2014; Couture, Penn, & Roberts, 2006), alexithymia may contribute to these difficulties through atypical judgments of others. Measuring, reporting, and controlling for levels of co-occurring alexithymia should therefore be routine practice when studying character inferences in clinical populations. We note that published reports of trait judgments in ASD have already yielded considerable inconsistency (e.g., Caulfield, Ewing, Burton, Avard, & Rhodes, 2014; Mathersul, McDonald, & Rushby, 2013; Pinkham, Hopfinger, Pelphrey, Piven, & Penn, 2008). We speculate that where observed, atypical inferences in this population may be due to co-occurring alexithymia.

The current results present evidence for the impact of alexithymia on emotion-mediated trait inferences, but it could be argued that trait inferences are further influenced by theory of mind (ToM; the ability to represent others’ mental states) in real-world social situations. Whether alexithymia is accompanied by ToM deficits is currently a matter of debate (Bernhardt et al., 2014; Moriguchi et al., 2006; Silani et al., 2008; Subic-Wrana, Beutel, Knebel, & Lane, 2010; Wastell & Taylor, 2002). Although it is unlikely that the static faces used in this study prompted mental state attributions, any ToM deficit related to alexithymia may impact trait attributions in more ecologically valid settings as a result of atypical mental state attributions. This possibility should be a target of future study.

It is of note that individuals with alexithymia did not differ from control individuals in their inferences of happiness from neutral faces. Although alexithymia impairs recognition of a broad range of emotions, some evidence suggests intact positive emotion recognition in individuals with alexithymia (McDonald & Prkachin, 1990). Although this is not necessarily the case (see Brewer, Cook, Cardi, Treasure, & Bird, 2015), it may be true that alexithymia does not impede detection of positive cues to the same degree as detection of negative cues. This possibility is difficult to determine in the current study, as interrater reliability of happiness judgments from neutral faces does not equate to objective recognition of happiness from expressive faces. As inference of a number of traits has been found to rely on detection of happiness cues, however, the impact of potentially typical happiness recognition in individuals with alexithymia is of interest. It may be the case that individuals with alexithymia make trait judgments that rely most strongly on happiness recognition in a typical way. Indeed, intelligence and attractiveness judgments, which rely strongly on cues of happiness, were relatively typical in the current experiment. Determining whether intact recognition of happiness cues in alexithymia impacts trait judgments strongly associated with happiness therefore remains a priority for future work.

In conclusion, the present findings provide strong support for the emotion-overgeneralization hypothesis of character inference. That individuals with developmental deficits of emotion recognition exhibit atypical trait judgments suggests that mechanisms of emotion recognition play a causal role in the inference of character traits. Clearly, the use of emotional cues to judge character traits is a universal phenomenon, unaltered by one’s emotion-recognition abilities. Impoverished recognition of facial emotion, and atypical inferences about the character of others, may have profound consequences for the social interactions of individuals with alexithymia.
